# A Crucial Role of Bone Morphogenetic Protein Signaling in the Wound Healing Response in Acute Liver Injury Induced by Carbon Tetrachloride

**DOI:** 10.1155/2012/476820

**Published:** 2012-06-03

**Authors:** Nao Oumi, Kumiko Amano Taniguchi, Ayumi Miyashita Kanai, Mayu Yasunaga, Tomoko Nakanishi, Kenzo Sato

**Affiliations:** ^1^Department of Molecular Biology, School of Life Sciences, Faculty of Medicine, Tottori University, Nishi-cho, Yonago 683-8503, Japan; ^2^Tottori University Chromosome Engineering Research Center, Nishi-cho, Yonago 683-8503, Japan

## Abstract

*Background.* Acute liver injury induced by administration of carbon tetrachloride (CCl_4_) has used a model of wound repair in the rat liver. Previously, we reported transient expression of bone morphogenetic protein (Bmp) 2 or Bmp4 at 6–24 h after CCl_4_ treatment, suggesting a role of BMP signaling in the wound healing response in the injured liver. In the present study, we investigated the biological meaning of the transient *Bmp* expression in liver injury. *Methods.* Using conditional knockout mice carrying a floxed exon in the BMP receptor 1A gene, we determined the hepatic gene expressions and proliferative activity following CCl_4_-treated liver. *Results.* We observed retardation of the healing response in the knockout mice treated with CCl_4_, including aggravated histological feature and reduced expressions of the *albumin* and *Tdo2* genes, and a particular decrease in the proliferative activity shown by Ki-67 immunohistochemistry. *Conclusion.* Our findings suggest a crucial role of BMP signaling in the amelioration of acute liver injury.

## 1. Introduction

The mammalian liver is the main organ for metabolism of nutrients and drugs, as well as storage of glycogen and lipids, synthesis and secretion of serum proteins, as well as detoxification, and production of biochemicals necessary for digestion [1, 2]. Moreover, the liver has a robust ability to self-regenerate from the remaining tissue after two-third partial hepatectomy [3, 4]. Chronic liver injury caused by hepatitis viruses, autoimmune responses, hepatotoxin intake, or cholestatic and metabolic diseases progresses to liver cirrhosis or fibrosis through stimulation of quiescent hepatic stellate cells to proliferate and transform into fibroblast cells [5]. However, the earliest stage of these processes is thought to consist of repeated cycles of injury and repair in liver cells [6, 7]. Acute liver injury can be caused by various pharmacological toxicants. A dynamic regeneration or tissue repair response similar to that after partial hepatectomy occurs following cell death and tissue injury caused by exposure to toxic chemicals. An intricate signal transduction network consisting of chemokines, cytokines, growth factors, and hormones has been revealed for liver regeneration after partial hepatectomy [8, 9].

Acute liver injury induced by carbon tetrachloride (CCl_4_) is widely studied as a model of liver injury in rats. CCl_4_ is metabolized by cytochrome P450 IIE1 [10] in mature hepatocytes and converted to trichloromethyl radicals, resulting in acute but reversible damage to the centrilobular hepatocytes that is followed by liver regeneration. Recently, we observed that BMP2 or BMP4 were transiently expressed in the early stage of CCl_4_ injury in rats [11]. However, the role of Bmp2 or Bmp4 in CCl_4_ liver injury was not clarified [11]. Bmp2 and Bmp4 are members of the transforming growth factor- (TGF-) **β** superfamily and are involved in the development of many organs, including the liver. In hepatogenesis, Bmp2 is secreted from the cardiac mesoderm and participates in morphogenetic growth of the hepatic endoderm into a liver bud. In mice, Bmp4 has an important role in hepatogenesis in early embryos [12, 13]. Therefore, BMPs are thought to be involved in cell proliferation and determination of progenitor cell fate.

BMP2 and BMP4 signals are transduced by hetero-multimers of two types of transmembrane serine/threonine kinases, Bmp type 1A and type 2 receptors (*Bmpr1a* and Bmpr2, resp.). Mice homozygous for a *Bmpr1a*-null allele die at embryonic day 8.0 without mesoderm formation [14]. In the present study, to examine whether the BMP signaling transiently expressed in CCl_4_ liver injury is involved in liver regeneration, we investigated the liver injury and healing in *Bmpr1a* conditional knockout (*Bmpr1a*-KO) mice induced by the *Cre*/*lox*P system [15, 16]. In these mice, intravenous injection of a recombinant *Cre* adenovirus efficiently induces transgene expression in most of liver cells, and genomic knockout of the *Bmpr1a *gene is specifically induced in the liver. If BMP signaling serves as a regenerative cue in the early stage of liver injury, *Bmpr1a*-KO mice should exhibit retarded restoration of liver function.

## 2. Materials and Methods

### 2.1. Ethics Statement

All of the animal experiments described were approved by the Institutional Animal Care and Use Committee of Tottori University (permission numbers: 18-2-39 and 06-S-80). All the mice received humane care in compliance with Tottori University's guidelines for the care and use of laboratory animals in research.

### 2.2. Animals

The mice in this study were fed ad libitum and housed in a room maintained at a constant temperature of 22°C, with 50% humidity and a 12-h/12-h light/dark cycle. Eight-week-old male ICR mice were purchased from Nihon Clea (Tokyo, Japan). *Bmpr*1*a* ^+/−^ mice and *Bmpr*1*a* ^flox/flox^ mice were obtained from Dr. Yuji Mishina (National Institute of Environmental Health Sciences, Research Triangle Park, NC; present in University of Michigan School of Dentistry) [15, 16]. Male *Bmpr*1*a* ^flox/−^ mice weighing 30 g were generated by breeding between *Bmpr*1*a* ^+/−^ and *Bmpr*1*a* ^flox/flox^ mice and used at 10 weeks of age. The genotypes of the mice were determined by PCR of genomic DNA. The following primer sets were used: flox allele detection, forward 5′-GCAGCTGCTGCTGCAGCCTCC and reverse 5′-TGGCTACAATTTGTCTCATGC; null allele detection, forward 5′-AGACTGCCTTGGGAAAAGCGC and reverse 5′-GGACTATGGACACACAATGGC.

### 2.3. Biochemical Measurements

The aspartate aminotransferase (AST) and alanine aminotransferase (ALT) activities in serum samples from the treated mice were determined using L-Type WAKO AST/ALT J2 assay kits (Wako Pure Chemicals Co. Ltd., Osaka, Japan), according to the manufacturer's instructions.

### 2.4. Recombinant Adenovirus

A recombinant adenovirus vector (Ad-*Cre*) was constructed with human adenovirus type 5 by replacing the *E1A* and *E1B* genes with the bacteriophage P1 *Cre* recombinase gene under the control of the CAG promoter. Likewise, Ad-*LacZ* was constructed with the *Escherichia coli LacZ* gene as a control. The recombinant adenoviruses were propagated in HEK293 cells, which are human embryonic kidney cells transformed by the *E1A* and *E1B* genes. Virions were purified by CsCl equilibrium centrifugation, dialyzed against 10 mM HEPES containing 1 mM EDTA and 10% glycerol, and titrated with HEK293 cells.

### 2.5. CCl_4_ Injury and Infection of the Adenovirus In Vivo

ICR mice were treated with 5 *μ*L of CCl_4_/liquid paraffin (1 : 4 mixture) per gram of body weight and euthanized at 24 or 72 h postinjection for total RNA extraction. *BMPR*1*A* ^flox/−^  mice were infected with 100 *μ*L (1.5 × 10^8^ pfu) of the purified recombinant adenovirus Ad-*Cre* or Ad-*LacZ* via the tail vein by single injection. Mock-infected mice were injected with phosphate-buffered saline (PBS). The infected mice were treated with CCl_4_ at 14 days postinfection. Subsequently, the mice were euthanized after 24 or 72 h to obtain livers for tissue sections and genomic DNA and total RNA isolation.

### 2.6. RNA Preparation, RT-PCR, and Real-Time PCR

Total RNA was isolated from mouse tissues by acid phenol-guanidinium thiocyanate-chloroform extraction. Total RNA (2 *μ*g) was converted to complementary DNA and the target genes were amplified using Taq DNA polymerase (Bio Academia, Osaka, Japan) in a PCR thermal cycler using the primer sets as follows: Bmp2: 5′-GACGGACTGCGGTCTCCTAAAG and 5′-TCTGCAGATGTGAGAAACTCGTCA, Bmp4: 5′-GAGGAGTTTCCATCACGAAGA and 5′-GCTCTGCCGAGGAGATCA, *Bmpr1a*: 5′-GAAAGCAGCAGGTGAAAGTC and 5′-CTATAATGGCAAAGCAATGG, Id1: 5′-GGATCATGAAGGTCGCCAGT and 5′-TTGCTCACTTTGCGGTTCTG, Id2: 5′-GGTCTTCCTCCTACGAGCAG and 5′-ACGATAGTGGGATGCGAGT, Id3: 5′-AGCTCACTCCGGAACTTGTG and 5′-GGGACAGAGTGACGTTGCC, Albumin: 5′-GAAGACCCCAGTGAGTGAGC and 5′-CAGTCGAGAAGCAGGTGTCC, AldolaseB: 5′-ATTTCATTGTCTTTGCCTAT and 5′-ATGCCAAGTCAGGTTTATCA, Tdo2: 5′-AAGGTGAACGACGACTGTCA and 5′-AGTTGAACGCAGGTAATGAT, PEPCK: 5′-GACCCTTCTTCGGCTACAAC and 5′-CTGGATTCCTGAGTGACCTT, Transferrin: 5′-CGGGTTAAGGCTGTACTGAC and 5′-TAAGGCACAGCAGCGAAGAC, PCNA: 5′-CTTACTCTGCGCTCCGAAGG and 5′-CAAATTCACCCGACGGCATC, GAPDH: 5′-AAGGCTGTGGGCAAGGTCAT and 5′-CACCACCCTGTTGCTGTAGC. Quantitative analyses were also performed to measure the mRNA levels by real-time PCR (ABI 7900HT; Applied Biosystems Co., Foster City, CA) with TaqMan probes (Applied Biosystems Co.) according to the manufacturer's protocol.

### 2.7. Immunohistochemistry

Livers were fixed in 10% formalin and embedded in paraffin. After deparaffinization in xylene and rehydration in a graded ethanol series, 7-*μ*m sections were immersed in a vessel containing 10 mmol/L citrate buffer (pH 7.0) and autoclaved at 121°C for 15 min. The sections were then treated with 3% (v/v) H_2_O_2_ for 10 min at room temperature, blocked with 10% (v/v) goat serum or rabbit serum (Nichirei, Tokyo, Japan) for 30 min at room temperature, and incubated with a rabbit monoclonal antibody against Ki-67 (Thermo Fisher Scientific Inc., San Jose, CA) diluted 1 : 200 for 1 h at room temperature. After washing with PBS, the sections were incubated with biotinylated goat anti-rabbit IgG (Nichirei) or biotinylated rabbit anti-mouse IgG (Nichirei) for 30 min. The sections were washed with PBS, incubated with a solution of streptavidin-conjugated horseradish peroxidase (Nichirei) for 15 min according to the manufacturer's recommendations and washed again with PBS for 5 min. Peroxidase activity was detected with H_2_O_2_/diaminobenzidine substrate solution and the sections were counterstained with hematoxylin before dehydration and mounting. The percentage of Ki-67-positive hepatocytes was determined by counting positively stained hepatocyte nuclei in 40 random fields at 40× magnification and calculating the mean value. The value was expressed as a fraction of the total number of hepatocytes in a 40× field, which averaged 30 cells/field.

### 2.8. *β*-Galactosidase Staining

Determination of the expression of the lacZ gene was carried out according to Jaffe et al. [17]. Briefly, the fixed specimens were rinsed three times with PBS and incubated in a reaction mixture containing 5 mM KaFe(CN)6, 5 mM K4Fe(CN)6, 2 mM MgCI2, and 1 mg/mL X-gal (5-bromo-4-chloro-3-indolyl-*β*-D-galactopyranoside) in PBS for 2 h at 37°C. Subsequently, these specimens were counterstained with eosin.

### 2.9. Immunofluorescence

Liver sections prepared as in immunohistochemistry were blocked with 10% (v/v) goat serum (Nichirei, Tokyo, Japan) for 30 min at room temperature and incubated with a rabbit polyclonal antibody against *BMPR1A* (ABGENT Inc, San Diego, CA) diluted 1 : 50 for 1 h at room temperature. After washing with PBS, the sections were incubated with Alexa fluor 488 conjugated goat anti-rabbit IgG (Invitrogen, Austin, TX) diluted 1 : 1000 for 1 h at room temperature. The sections were washed with PBS and counterstained with DAPI before mounting. Images were acquired with OLYMPUS Laser Confocal Scannig Microscope FV1000D Spectral Type (inverted microscope *I* × 81).

### 2.10. Statistical Analysis

Statistical analysis was performed using StatView (SAS Institute Inc., Cary NC). The Student's *t*-test was used to analyze the difference between the study and control groups; *P* values less than 0.05 were considered statistically significant.

## 3. Results

### 3.1. Transient Expression of BMP4 in Mice Treated with CCl_4_

**Figure 1:**
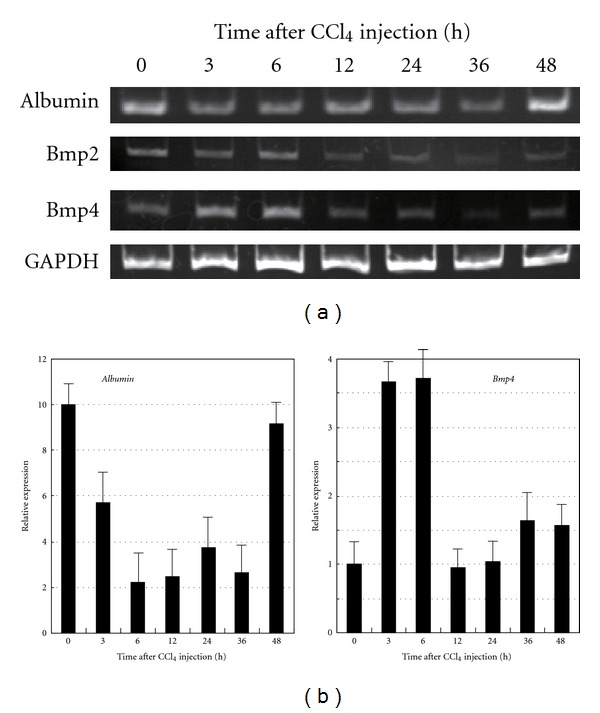
Time course of gene expressions in the liver of mice treated with CCl_4_. (a) RT-PCR analyses of BMP2, BMP4 and albumin were performed using the primer sets shown in Section 2. Albumin expression is decreased at 3–36 h and recovers at 48 h. BMP4 is transiently expressed at 3–6 h after CCl_4_ injection. (b) Real-time PCR analyses of *albumin* and *BMP4* were performed using primer sets and TaqMan probes provided by Applied Biosystems Co. All data are shown as the means ± SE from three independent experiments.

Previously, we reported that *Bmp2 *or *Bmp4* are transiently expressed in CCl_4_-treated rats in the early stage of liver injury [11]. To confirm whether this transient expression of *Bmps* is also observed in mice treated with CCl_4_, we examined the expressions of liver-specific genes and the *Bmp2 *or *Bmp4 *genes in the liver-injured mouse model by RT-PCR and real-time RT-PCR. Albumin mRNA expression, which was examined as a marker for liver function, was decreased at 3–36 h and recovered at 48 h after treatment with CCl_4_ (Figures 1(a) and 1(b)). Likewise, we determined the expressions of the *Bmp2 *or *Bmp4* genes in liver-injured mice, because *Bmps*, especially *Bmp4*, play an important role in liver development during mouse embryogenesis. *Bmp4* mRNA was significantly and transiently induced at 3–6 h after treatment with CCl_4_, while *Bmp2* mRNA was slightly induced (Figures 1(a) and 1(b)). These findings are similar to the wound and repair responses in the liver injury model in rats, and they suggest that Bmp4 is also involved in the wound healing response in the injured liver of mice.

### 3.2. *Bmpr1a* Knockout in Liver by *Cre* Recombinase

**Figure 2:**
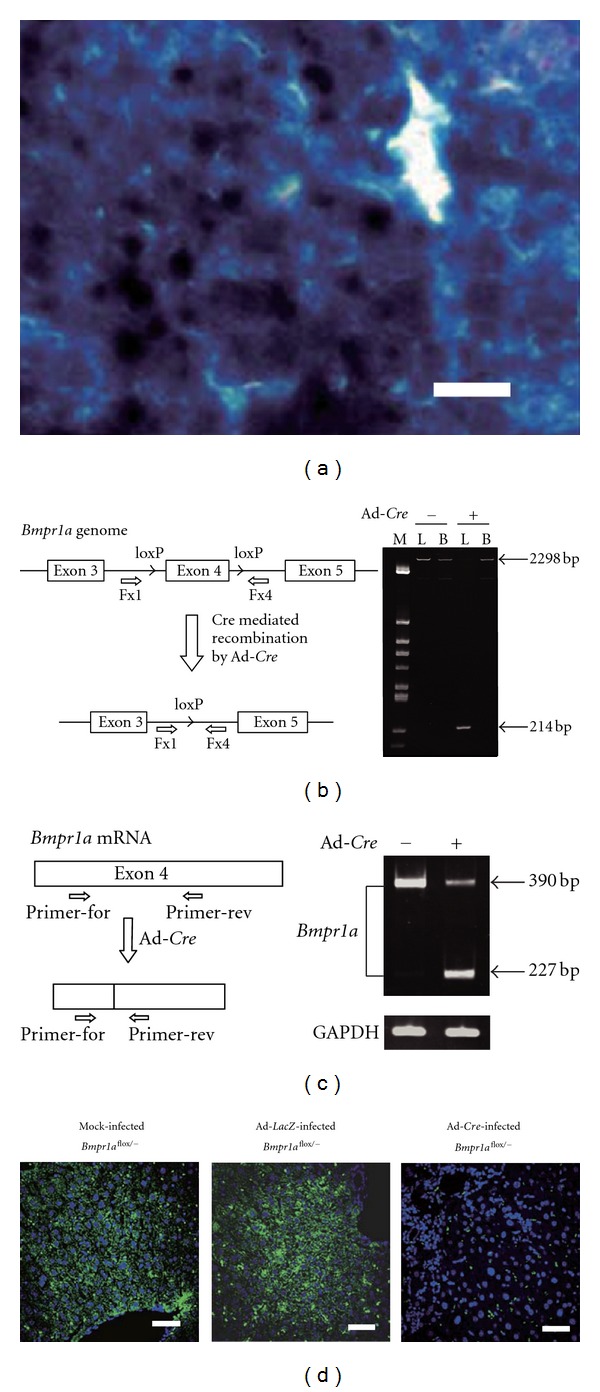
Generation of *Bmpr1a *knockout mouse with *Cre* recombination system (a) *β*-galactosidase staining of liver from Ad-*LacZ*-infected mouse. Scale bars: 50 *μ*m. (b) Left panel: schematic illustration of floxed *Bmpr1a* gene and generation of deletion. Right panel: genomic PCR. Genomic DNA was obtained from liver (L) and brain (B) at 14 days postinfection (Ad-*Cre* +). PCR was performed using primer set: 5′-GGTTTGGATCTTAACCTTAGG (Fx1)/5′-TGGCTACAATTTGTCTCATGC (Fx4). (c) Left panel: schematic illustration of transcripts from *Bmpr*1*a* ^flox^ and *Bmpr*1*a* ^−^  genes generated by *Cre* recombination. Right panel: total RNA was obtained from the liver at 14 days postinfection with Ad-*Cre*. RT-PCR for *BMPR1A* transcripts was carried out with primer sets: 5′-GAAAGCAGCAGGTGAAAGTC (Primer-for)/5′-CTATAATGGCAAAGCAATGG (Primer-rev). RT-PCR of GAPDH mRNA was performed as a control. (d) Imunofluorescence of *Bmpr1a* in the livers from mock- Ad-LacZ- and Ad-*Cre*-infected *Bmpr*1*a* ^flox/−^ mice. Strong signals without nuclei were derived from erythrocytes Scale bars: 25 *μ*m.

To determine the role of Bmp4 in acute liver injury, we analyzed the wound healing response in injured conditional knockout mice with inhibited Bmp4 signaling by deletion of *Bmpr1a*. Since *Bmpr1a*-null mice (*Bmpr*1*a* ^−/−^) show embryonic lethality [14], we used *Bmpr1a*-floxed mice in which both sides of exon 4 in the *Bmpr1a* gene were flanked by *lox*P sites and generated liver-specific *Bmpr1a* knockdown using the Ad-*Cre* adenovirus expressing *Cre* recombinase. Removal of exon 4 of the *Bmpr1a* gene is known to delete the biological function of *Bmpr1a* [16]. To obtain complete deletion of the *Bmpr1a* gene in the mouse genome, we generated *Bmpr*1*a* ^flox/−^ heterozygotic mice by mating between *Bmpr*1*a* ^flox/flox^ mice and *Bmpr*1*a* ^+/−^ mice. It is known that an adenovirus can efficiently infect liver cells through blood circulation from a peripheral vein [18]. First, we confirmed that most of liver cells are efficiently infected with adenovirus vectors carrying LacZ gene by intravenous injection (Figure 2(a)). As the result, single injection of Ad-*Cre* into the floxed mice induced deletion of the* Bmpr1a* gene in *Bmpr*1*a* ^flox/−^ mice livers. The wild-type (or floxed) *Bmpr1a *gene is 2298 bp in length in genomic PCR, while the deleted allele is 214 bp in length (Figure 2(b)). *Cre*-mediated recombination occurred in the liver. Furthermore, when exon 4 of the *Bmpr1a* gene was excised from the genome by *Cre* recombinase, the shortened mRNA lacking exon 4 should be transcribed. The RT-PCR product is 390 bp before recombination and 227 bp after recombination. Expression of *Bmpr1a* mRNA lacking exon 4 was confirmed in Ad-*Cre*-injected mice (Figure 2(c)). Furthermore, expression of Bmpr1a protein was significantly decreased in Ad-*Cre*-infected mouse liver (Figure 2(d)). Small and strong signals without nuclei in each panels were derived from erythrocytes remained in liver tissue. These results indicate that *Cre*-mediated recombination resulted in the removal of the *Bmpr1a* gene from the liver of Ad-*Cre*-injected mice.

### 3.3. Liver Injury in *Bmpr1a*-KO Mice

**Figure 3:**
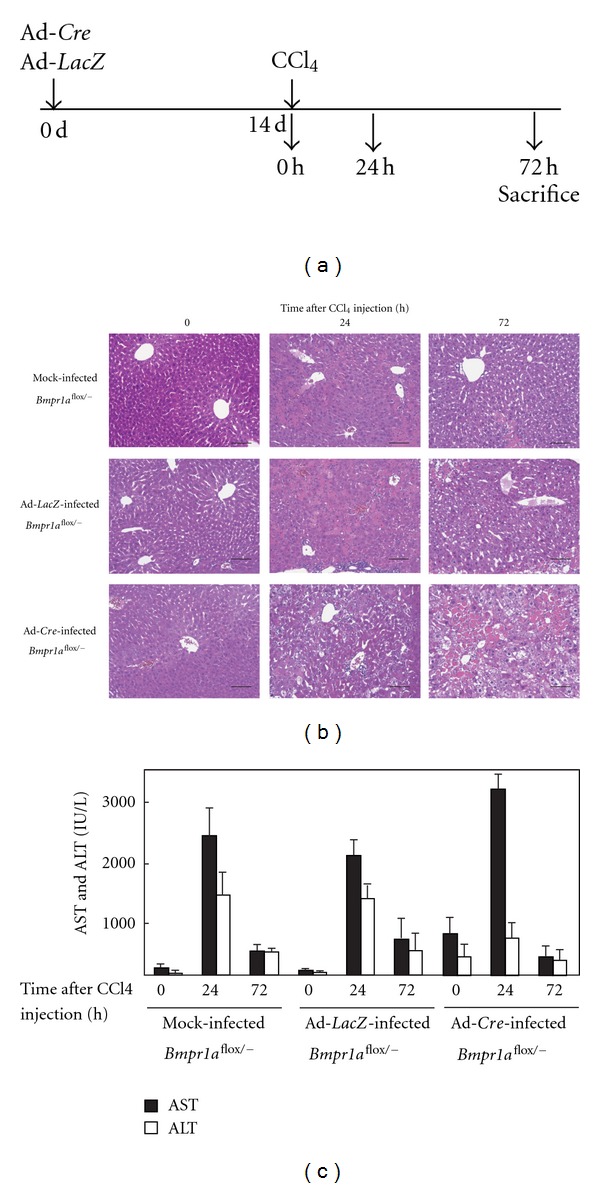
Liver injury in *BMPR1A*-KO mice. (a) Time course for the experiment. *BMPR*1*A* ^flox/−^ mice were infected with mock, Ad-*Cre* or Ad-*LacZ* for 14 days, followed by intraperitoneal administration of CCl_4_ for the indicated time. (b) Hematoxylin and eosin staining of tissue sections from the injured liver in *BMPR*1*A* ^flox/−^ mice. The hepatotoxicity of CCl_4_ causes necrotic damage to the centrilobular hepatocytes at 24 h. The recovery from the liver injury in *BMPR1A*-KO mice is retarded at 72 h after CCl_4_ injection compared with control mice. Scale bars: 50 *μ*m. (c) AST activities in serum samples from CCl_4_-treated mice. All data are shown as the means ± SE from three independent experiments.

To determine whether BMP signaling is involved in the wound healing response in liver injury, we induced CCl_4_ liver injury in *Bmpr1a*-KO mice generated by single injection of Ad-*Cre* for 14 days when inflammatory response by adenovirus infection should be cured (Figure 3(a)). The extent of the liver injury was determined histologically by hematoxylin and eosin staining of tissue sections. In the control mock-infected *Bmpr*1*a* ^flox/−^ mice, severe damage to the centrilobular hepatocytes was observed at 24 h after CCl_4_ injection, and most of the necrotic hepatocytes had disappeared at 72 h (Figure 3(b)). Similar observations were noted in the injured liver of *Bmpr*1*a* ^flox/−^ mice infected with Ad-*LacZ* as a control (Figure 3(b)). In contrast to these control mice, *Bmpr*1*a* ^flox/−^ mice infected with Ad-*Cre* (*Bmpr1a*-KO mice) showed a low level of amelioration of the injured liver histologically at 72 h after CCl_4_ injection (Figure 3(b)). On the other hand, the serum AST and ALT activities of the mice were increased at 24 h after CCl_4_ treatment and recovered to the basal levels at 72 h in both the control and knockout mice (Figure 3(c)). However, AST activity in KO mice was shown at little bit high level compared to Mock and Ad-*LacZ* infected mice without CCl_4_ by unknown reason. These findings suggest that a single injection of CCl_4_ induced transient liver damage regardless of the presence or absence of BMP signaling, but it did not induce additional damage. However, amelioration of the wound healing response was dependent on the presence of BMP signaling.

**Figure 4:**
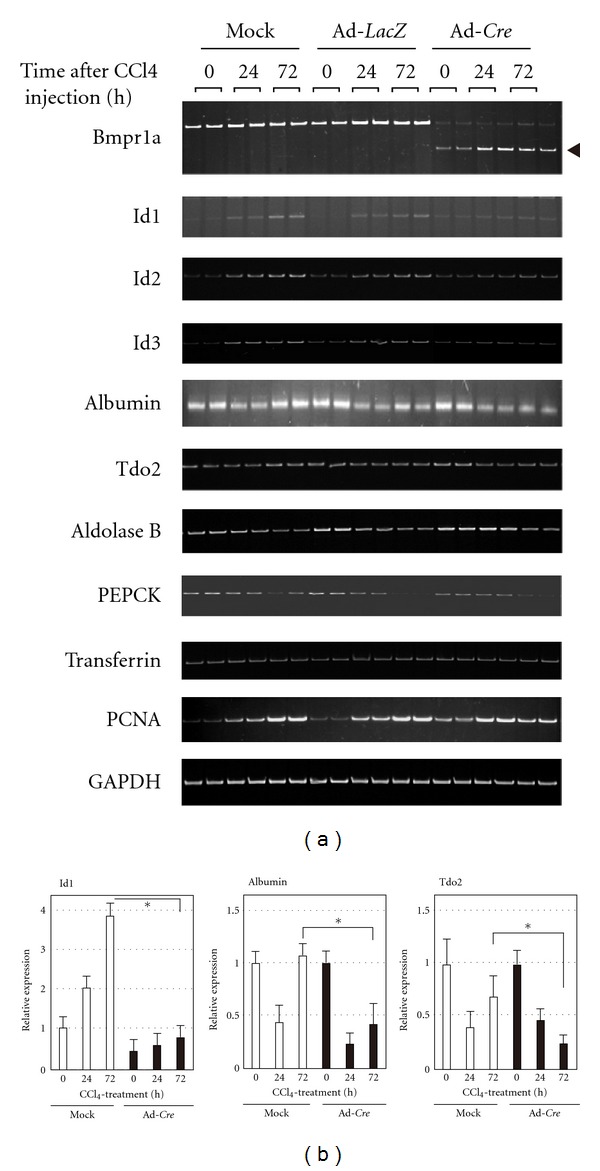
Gene expressions in the CCl_4_-injured liver of *BMPR1A*-KO mice. (a) RT-PCR was performed in duplicate with total RNA from CCl_4_-injured livers of *BMPR*1*A* ^flox/−^ mice infected with mock, Ad-*LacZ* or Ad-*Cre.* Short size PCR products in *BMPR1A* (arrowhead in top panel) shows the deletion in *BMPR1A* mRNA generated by *Cre* recombination. (b) Real-time RT-PCR of Id1, albumin, and Tdo2. Relative expression was shown by ratio of albumin expression levels normalized by *β*-actin internal control to the value in 0-time of CCl_4_-treated liver. All data are shown as the means ± SE from three independent experiments. *P < 0.05, significant difference by Student's *t*-test.

To determine whether BMP signaling is involved in the wound healing response at the molecular level in the CCl_4_ liver injury model, we examined the expressions of various hepatic genes in *Bmpr1a*-KO mice by RT-PCR. Initially, we evaluated the expression of *Bmpr1a* mRNA carrying exon 4 deletion after Ad-*Cre* infection. As shown in Figure 4(a), a shorter *Bmpr1a* mRNA (shown with arrowhead) was expressed in the liver of *Bmpr1a*-KO mice, while the full-length *Bmpr1a* mRNA was observed in both mock-infected and Ad-*LacZ*-infected *Bmpr*1*a* ^flox/−^ mice. Interestingly, the levels of *Bmpr1a* expression were less affected by CCl_4_ treatment. Furthermore, the expressions of *Id1*, *Id2,* and *Id3*, as target genes for BMP signaling, were induced by CCl_4_ treatment for 24 and 72 h in mock-infected and Ad-*LaxZ*-infected *Bmpr*1*a* ^flox/−^ mice, but they were significantly reduced in *Bmpr1a*-KO mice (Figure 4(a)). These observations are consistent with our finding that *Bmp4* was induced by CCl_4_ treatment.

To evaluate the hepatic function in the CCl_4_-treated liver of *Bmpr1a*-KO mice, we determined the expressions of hepatic genes by RT-PCR (Figure 4(a)). The reduced expression of the albumin gene in the injured liver was increased at 72 h posttreatment with CCl_4_ in mock-infected and Ad-LacZ-infected *Bmpr*1*a* ^flox/−^ mice as controls, whereas little restoration was observed in *Bmpr1a*-KO mice. Similarly, the expressions of the *aldolase B* and tryptophan 2,3-dioxygenase (*Tdo2*) genes in *Bmpr1a*-KO mice were hardly recovered, compared with control mice. These observations were confirmed by quantitative real-time RT-PCR (Figure 4(b)). Meanwhile, the reduced expression of the *aldolase B* and phosphoenolpyruvate carboxykinase (*Pepck*) gene by CCl_4_ treatment was not restored in either the control mice or *Bmpr1a*-KO mice. Moreover, the expression of the transferrin gene was barely or not influenced by CCl_4_ treatment and the absence of BMP signaling. These findings show that some of the hepatic gene expressions reduced by the hepatotoxin were recuperated through BMP signals expressed in the wounded liver, suggesting a role for the transient expression of *Bmp4*.

### 3.4. Decreased Hepatocyte Proliferation in *Bmpr1a*-KO Mice

**Figure 5:**
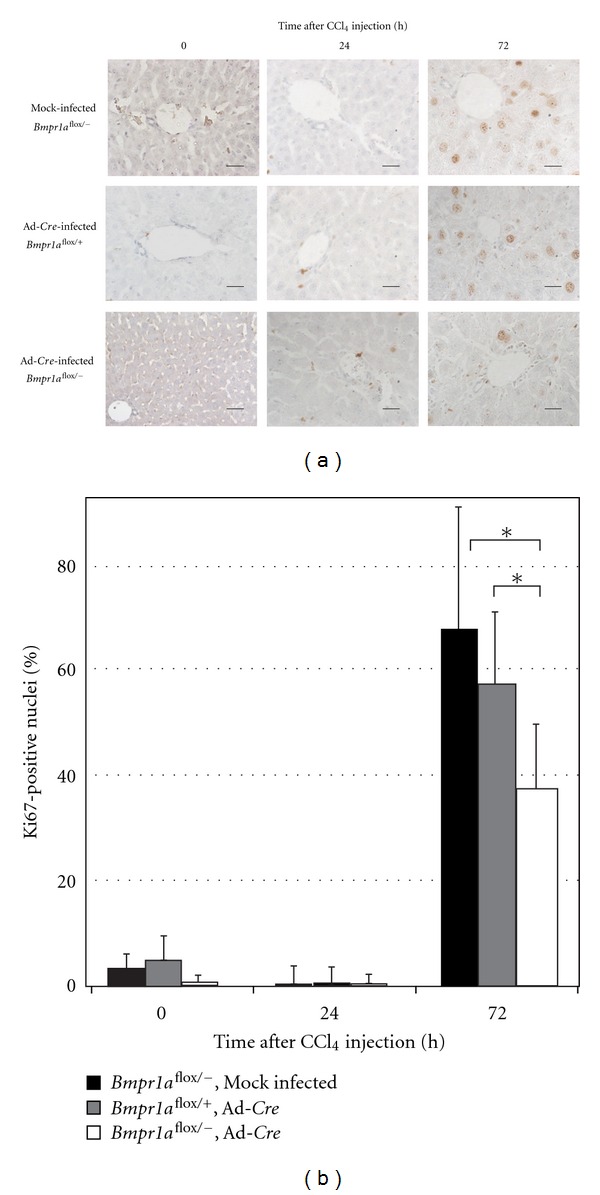
Proliferative activity in the CCl_4_-injured liver of *BMPR1A*-KO mice. (a) Immunohistochemistry for Ki-67, a cell cycle marker, in the CCl_4_-injured liver. *BMPR*1*A* ^flox/−^ mice were infected with mock or Ad-*Cre* for 14 days, and *BMPR*1*A* ^flox/+^ mice were infected with Ad-*Cre* as a control. Brown nuclei indicate Ki-67-positive cells. Scale bars: 25 *μ*m. (b) Quantitative analysis of Ki-67-positive cells. Ki-67-positive cells were counted in 10 randomly taken microscopic photos, and are shown as percentages relative to the total cell number. All data are shown as the means ± SE from three independent experiments. *P < 0.05, significant difference by Student's *t*-test.

Hepatocyte proliferation and differentiation are necessary for the healing process after liver injury. We determined the proliferative activity in the injured liver by evaluating the expression of proliferating cell nuclear antigen (PCNA) by RT-PCR (Figure 4(a)) and the cell cycle marker Ki-67 by immunohistochemistry (Figure 5). *Bmpr1a*-KO mice treated with CCl_4_ showed significantly decreased expression of *Pcna* at 24 and 72 h post-treatment, compared with mock-infected and Ad-*LacZ*-infected mice. Furthermore, Ki-67-positive cells were markedly fewer in number in the liver of *Bmpr1a*-KO mice at 72 h after CCl_4_ treatment, while the number of proliferating cells was increased in the liver of control mice after CCl_4_ injection (Figure 5). These findings indicate that BMP signaling was involved in the cell proliferation during the wound healing response in the CCl_4_-injured liver.

## 4. Discussion

Chronic liver diseases are aggravated by repeated cycles of injury and repair in liver cells [7, 19]. Therefore, understanding the mechanism and regulation of the elementary processes in the wound healing response may lead to novel therapeutic methods for these liver diseases. In this study, we observed that a single injection of CCl_4_ into mice induced transient expression of *Bmp4*, which is involved in hepatogenesis in early embryos. This finding suggests that the processes involved in liver development are tightly associated with the repair of acute liver injury. BMP4 is also involved in hepatogenesis, while Bmp7 was reported to facilitate regeneration of the injured kidney [20].

Previously, we reported that *Bmp2 *or *Bmp4* were induced in hepatocyte progenitor or oval-like cells, but not in Kupffer or macrophage cells, during liver injury [11]. Oval cells are hepatic stem-like cells (progenitor cells) derived from bone-marrow cells [21–25]. The mechanism underlying the induction of *Bmp4* expression after liver injury remains unknown, and it needs to be clarified. In this study, we have shown a crucial role of BMP signaling in the proliferation and differentiation of hepatic cells, including progenitor cells, in the response to liver injury induced by CCl_4_ using *Bmpr1a*-KO mice. In the early stage of embryonic development, Bmp2 or Bmp4 derived from the cardiac mesoderm or septum transversum mesenchyme are required for morphogenetic movement of the liver bud, including hepatic competence and endodermal patterning in the foregut ventral endoderm expressing *Gata-4* [12, 26]. Therefore, we can consider that the BMP signaling in the wound healing response to liver injury in adult rodents may imitate hepatogenesis in the early embryo.

Previously, we showed that hepatic stem-like cells differentiate in a stepwise manner *in vitro* in response to a series of cytokines and extracellular matrix components, such as type I collagen, TGF-*β*, hepatocyte growth factor, and oncostatin M [27]. This process also mimics hepatocyte differentiation in the early step of embryogenesis. In the present study, we have shown a pivotal role of BMP signaling in the wound healing of acute liver injury, and also that hepatic genes such as *albumin* and *Tdo2* respond significantly to BMP signaling, although *aldolase B* and *Pepck* did not recover from the injury. Interestingly, *transferrin* gene expression was independent of the injury and BMP signaling. This observation suggested to us that the proliferation and differentiation of hepatocytes are regulated by BMP signaling partially or only in one of the steps. However, it still remains a possibility that the deletion in BMP signaling enhanced CCl_4_ injury by some metabolic alteration resulting in a delayed healing response.

Regarding the role of BMP signaling, our results are consistent with recent reports that regeneration in *Bmpr1a*-KO zebrafish is delayed after partial hepatectomy [28]. Furthermore, Id3, a target gene of BMP signaling, was reported to have an important role in the proliferation and differentiation of hepatoblasts during chick liver development [29]. Liver-specific knockout of the *Bmpr1a* gene after Ad-*Cre* infection is a very useful tool for elucidating the important role of BMP signaling in the wound healing response, and for the development of therapeutic protocols for hepatic disease based on the mechanism of the healing process.
